# Dietary diversity and possible sarcopenia among older people in China: a nationwide population-based study

**DOI:** 10.3389/fnut.2023.1218453

**Published:** 2023-06-30

**Authors:** Qiaoqiao Du, Yanhui Lu, Fan Hu, Xinglin Feng, Yunquan Zhang, Shaojie Li, Chi Zhang, Hua Zhang, Yi Zeng, Yao Yao, Zhaohui Lu, Wenya Zhang, Xiangyang Gao

**Affiliations:** ^1^Health Management Center, The Second Affiliated Hospital of Soochow University, Suzhou, China; ^2^Department of Endocrinology, The Second Medical Center & National Clinical Research Center for Geriatric Disease, Chinese PLA General Hospital, Beijing, China; ^3^Department of Health Policy and Management, School of Public Health, Peking University, Beijing, China; ^4^Department of Epidemiology and Biostatistics, School of Public Health, Wuhan University of Science and Technology, Wuhan, China; ^5^China Center for Health Development Studies, Peking University, Beijing, China; ^6^The Key Laboratory of Geriatrics, Institute of Geriatric Medicine, Chinese, Academy of Medical Sciences, National Center of Gerontology of National Health Commission, Beijing, China; ^7^Research Center of Clinical Epidemiology, Peking University Third Hospital, Beijing, China; ^8^Center for Healthy Aging and Development Studies, National School of Development, Peking University, Beijing, China; ^9^Health Management Institute, The Second Medical Center & National Clinical Research Center for Geriatric Diseases, Chinese PLA General Hospital, Beijing, China

**Keywords:** dietary diversity, possible sarcopenia, plant-based diet, older adults, China

## Abstract

**Background:**

Sarcopenia is a common geriatric disease. Many dietary factors may contribute to the development of sarcopenia. Few studies have been conducted on dietary diversity and sarcopenia in Chinese older adults. Among a nationwide sample, the objective of this study is to assess the association between the dietary diversity score (DDS) and the prevalence of possible sarcopenia. We considered the different patterns of dietary diversity in relation to possible sarcopenia.

**Methods:**

We conducted this analysis utilizing the cross-sectional data from the 2012, 2014, and 2018 waves of the Chinese longitudinal healthy longevity survey (CLHLS). A standard developed by the Asian Working Group for Sarcopenia 2019 (AWGS2019) was used to assess the possibility of sarcopenia. On the basis of the DDS generated by previous studies, we have constructed four new indicators as follows: total diet, animal-based diet, plant-based diet, and plant-based diet without the consumption of legume products and nuts. We used the generalized estimation equation (GEE) model to evaluate the associations between the DDS of the total diet, animal-based diet, plant-based diet, and plant-based diet without the intake of legume products and nuts and possible sarcopenia. These associations were statistically adjusted for a variety of potential confounders. Sensitivity analysis was performed by excluding some participants who were long-term bedridden, had Alzheimer's disease, or were terminally ill.

**Results:**

The analysis included 6,624 participants (mean age 83.4 years at baseline). In our study, we found that participants with a higher DDS of the total diet (OR = 0.62; 95% CI: 0.51–0.77), animal-based diet (OR = 0.62; 95% CI: 0.49–0.79), and plant-based diet (OR = 0.64;95% CI: 0.51–0.80) were at a lower risk of developing sarcopenia. In sensitivity analyses, the associations remained unchanged.

**Conclusion:**

Taking a diversified diet, including animal foods, may reduce the risk of developing sarcopenia. According to the findings of this study, adopting a diversified diet might reduce the risk of sarcopenia for older adults.

## 1. Introduction

Sarcopenia is a common geriatric disease characterized by loss of skeletal muscle mass, low muscle strength, and/or low physical performance and is a serious public health problem faced by aging societies in the current times (1–4). The prevalence of this disease in the elderly population is largely influenced by factors, such as gender, age, pathological conditions, and diagnostic criteria (4). The results of a systematic review indicate that the prevalence ranges from 0.2 to 86.5% (0.3–91.2% in women and 0.4–87.7% in men), using all classifications for sarcopenia (5). The most commonly used classifications were the European Working Group on Sarcopenia in Older People (EWGSOP; prevalence range: 0.4–57.4%) and the Asian Working Group for Sarcopenia (AWGS; prevalence range: 0.3–53.0%) (2, 5, 6). In a meta-analysis, the overall prevalence of sarcopenia among individuals older than 60 years was estimated at 10–27% (5). While it is generally associated with advanced aging, muscle loss begins as early as 40 years of age and affects the elderly as they experience changes in body composition, including a reduction in muscle mass, deterioration of muscle quality, and an increase in fat mass (5, 7). It is necessary to screen out people who may suffer from sarcopenia at an early stage (8).

There is growing evidence that sarcopenia is closely related to a variety of adverse outcomes, including falls, functional disability, frailty, and mortality (9, 10). In clinical practice, case-finding is warranted when a patient exhibits signs or symptoms of sarcopenia (i.e., feeling faint, walking slowly, falling, or having difficulty getting up from a chair) (9). In accordance with the recommendations of the International Conference on Sarcopenia and Frailty Research (ICFSR), subjects who screen positive for sarcopenia should be referred for further evaluation to confirm their diagnosis (10). However, the diagnosis of sarcopenia must be based comprehensively on three aspects as follows: muscle quality, muscle strength, and muscle function (2). Most of these methods are time-consuming, laborious, need to be carried out by professionally trained staff, and are unsuitable for large-scale population surveys (8). AWGS 2019 has defined a new concept of “possible sarcopenia” with the aim of facilitating timely lifestyle intervention in the primary healthcare setting and in preventive services and preventing further development of sarcopenia, which will contribute to greater awareness of sarcopenia prevention and interventions across diverse healthcare settings (2, 11). Based on the AWGS criteria, 29.6% of older adults (≥60 years) were diagnosed with possible sarcopenia from China Health and Retirement Longitudinal Study (CHARLS) (12). A cross-sectional study showed that 61% of Iranian elderly (≥60 years) living in the community were considered to have possible sarcopenia judging by handgrip strength according to the EWGSOP2 (13). Early screening for sarcopenia is not only helpful in identifying individuals who may suffer from sarcopenia at an early stage but can also provide references for the development and prognosis of other diseases (8).

Sarcopenia is a condition common in older people who are not physically active, do not exercise, and do not obtain sufficient nutrition. Numerous dietary factors may contribute to the development of sarcopenia, according to a wide range of evidence, such as inadequate protein intake, deficiency of vitamin D, and lack of long-chain polyunsaturated fatty acids and antioxidant nutrients (4, 9). Sarcopenia is characterized by malnutrition, particularly protein-energy malnutrition. Several previous studies have demonstrated that inadequate protein intake (both vegetables and animal proteins) negatively affects muscle protein synthesis in older people (14–17). When coupled with a monotonous dietary pattern, food intake declines by ~25% between the ages of 40 and 70 years, which may result in inadequate nutrient intake (4). Previous studies have focused on the association between food components or types and sarcopenia, making it difficult to identify complex interactions and synergistic effects (4, 18).

To address the limitations of previous studies, it is necessary to study the overall diet (18). Dietary diversity is defined as “the number of different foods or food groups consumed during a given reference period” and assessed by the dietary diversity score (DDS) (19). DDS usually entails assigning scores to different food and evaluating dietary diversity based on the sum of those scores, with higher scores indicating dietary variety. An increase in individual DDS is associated with increased dietary intake of macronutrients and micronutrients (20, 21). DDS is a well-recognized indicator for evaluating diet quality and nutrition of food security status (21). The DDS can be widely used in large-scale studies and surveys (such as the Demographic and Health Survey) as a convenient and inexpensive index for assessing nutrient adequacy and overall dietary quality (19). The National Health and Nutrition Examination Survey (NHANES) concluded that DDS could be viewed as a convenient and fast indicator for assessing dietary diversity and observed that improved diet quality was associated with increased dietary diversity (19, 22). Previous studies have additionally demonstrated an association between DDS and chronic diseases, such as cardiovascular diseases, cancer, diabetes, and metabolic syndrome, as well as its correlation with improved health status and prolonged longevity (21–23). According to data from several investigations, higher DDS was also associated with better cognitive function, fewer physical limitations, and lower psychological stress in the elderly (24, 25).

Several studies have reported an association between diet and sarcopenia (7, 13, 18, 26–29). There is, however, a lack of evidence from developing countries, as most of these studies were based on data from developed countries (23). While older people are at a higher risk of sarcopenia, few studies have been conducted still on dietary diversity and sarcopenia in Chinese setting, which has the world's largest older people population. Based on nationwide data, the objective of this study is to assess the association between DDS and the prevalence of possible sarcopenia in Chinese older adults and to provide fundamental information for establishing dietary guidelines for preventing sarcopenia in the population.

## 2. Materials and methods

### 2.1. Study population

**Figure 1:**
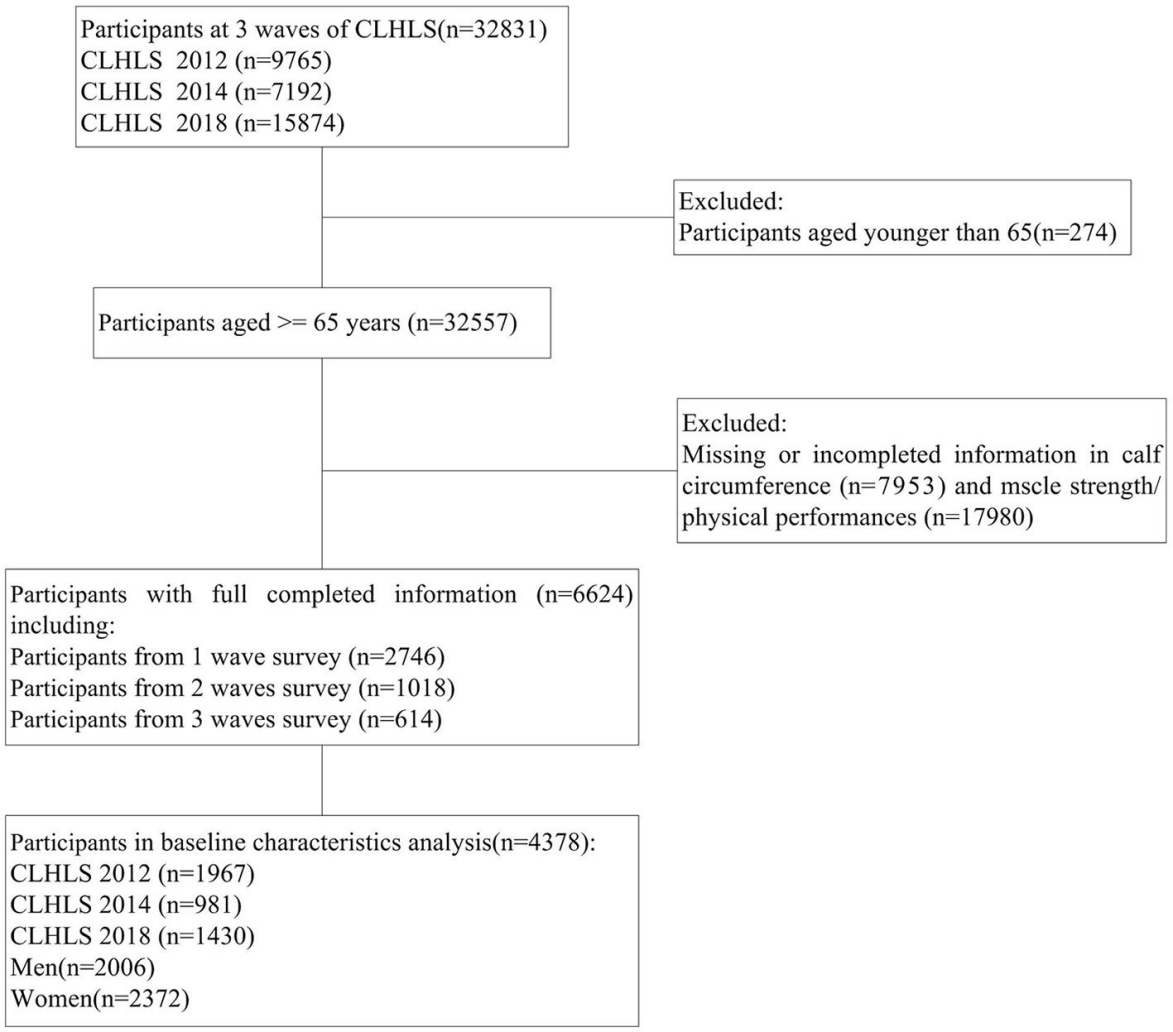
Flowchart of participants selection.

Data from the CLHLS were utilized in this study. CLHLS was conducted in 23 provinces out of the 31 provinces in China using a multistage, stratified cluster sampling design. Details of the sampling strategy have been discussed in our previous studies (30, 31). The association between DDS and possible sarcopenia was examined using data from the waves in 2012, 2014, and 2018. A total of 32,831 participants participated in the survey in 2012 (*n* = 9,765), 2014 (*n* = 7,192), and 2018 (*n* = 15,874). A total of 274 individuals aged below 65 years and 25,933 individuals with missing or incomplete information regarding calf circumference, muscle strength, or physical performance were excluded. In this study, 6,624 adults were enrolled in the final analytical sample (Figure 1). Informed consent was obtained from all participants and/or their families, and the study was approved by the Ethics Committee of Peking University (IRB00001052-13074).

### 2.2. Assessment of dietary diversity

The DDS was developed according to the dietary guidelines for Chinese populations which give suggestions regarding the consumption of 10 food groups (23). At the baseline, DDS was measured using a food frequency questionnaire relating to the 10 primary food groups, which included cereals, vegetables, fruits, legume products, nuts, meat, eggs, fish, dairy products, and funguses (23, 32). Those who consume cereals, vegetables, and fruits “every day/virtually every day or frequently” will receive one point for that food group. Funguses, legume products, nuts, meat, eggs, fish, and dairy products consumed “virtually every day or at least once a week” would be assigned one point. We calculated DDS for each participant with a total score of 10 and further assessed different dietary diversity by using the animal food group and the plant food group according to the previous research (33, 34). There are four main sources of animal-enriched diet food as follows: meat, fish, eggs, and dairy products. Using the frequency of intake of animal food, we calculated the DDS of the animal-based diet, which ranged from 0 to 4. There are six sources of plant-based food as follows: cereals, vegetables, fruits, legume products, nuts, and funguses. Our plant-based DDS ranged from 0 to 6, based on the frequency of plant-based food consumption. We scored low on DDS of a protein plant-based diet by using cereals, vegetables, fruits, and funguses on a scale of 0–4, excluding legume products and nuts.

### 2.3. Assessment of possible sarcopenia

To identify and diagnose older adults with or at risk of sarcopenia, AWGS2019 has developed a set of criteria, including case-finding, assessment, and diagnostic protocols suitable for use in hospitals, research settings, and in community healthcare and screening (2). A possible sarcopenia assessment was conducted using the AWGS2019 standards, which include three components as follows: calf circumference, muscle strength, or physical performance (2). The grip strength of the dominant hand and the non-dominant hand was measured as hard as possible in each hand. Muscle strength was determined by measuring the grip strength of both hands twice, utilizing a digital grip strength dynamometer. A maximum reading was obtained from at least two trials using either both hands or the dominant hand. In accordance with the recommendations of AWGS 2019, men and women were deemed to have low grip strength at a weight of 28 and 18 kg, respectively (2). AWGS 2019 recommends calf circumference < 34 cm for men and < 33 cm for women for screening or case-finding (2, 35). Physical performance was assessed with the question “how do you stand up after sitting on a chair?” Respondents were asked to select from three responses: “no problem,” “with problem,” and “not able to do it.” Responses of “with problem” or “not able to do it” indicated low physical performance (35).

### 2.4. Covariates

To obtain more reliable findings, we controlled for a broad set of potential confounders, which included gender (male or female), age groups (65–79, 80–99, and ≥100 years), education (0, 1–6, and ≥7 years), marital status (married/unmarried, divorced, or widowed), pre-retirement occupation (peasant or no-peasant), household income (< 100,000/≥100,000 yuan), smoking, drinking, and physical activity status (never/previous/current). Weight, height, and waist circumference were measured using standard anthropometric methods. Body mass index (BMI) was calculated as the weight in kilograms divided by the square of height in meters. Biochemical indices, including vitamin D3 and albumin, were obtained from the analysis of blood samples.

### 2.5. Statistical analysis

The data were presented as mean ± SD or percentages. Group comparisons used the chi-squared tests for qualitative variables and the independent *t*-test for quantitative variables. To fill in the missing covariates, multiple imputation methods were used. The odds ratios (ORs) of possible sarcopenia with the DDS quartile were calculated using generalized estimation equation (GEE) models (working correlation matrix structure: AR). Model 1 was an unadjusted model. Model 2 further controlled for gender, age, household income, level of education, marital status, and occupation before retirement. In Model 3, physical activities, smoking, drinking, BMI, and waist circumference were additionally controlled, and in Model 4, vitamin D3 and albumin were additionally controlled. To examine the robustness of the estimation, we performed a sensitivity analysis by excluding older adults who were long-term bedridden, had Alzheimer's disease, or were terminally ill. IBM SPSS statistics version 21.0 for Windows was used to analyze the data. Forest plots were performed using MedCalc version 20. Statistical significance was determined by a two-tailed *p*-value of 0.05.

## 3. Results

### 3.1. Characteristics of study participants

According to the sarcopenia status, the baseline characteristics of participants (*n* = 4,378) are shown in Table 1. The prevalence of possible sarcopenia was 66.9% (2,931/4,378). A total of 2,006 (41.8%) participants were men, 2,372 (58.2%) were women, and 1,835 (41.9%) were married. The participants were aged 83.4 ± 11.0 years, including 1,524 participants aged 65–79 years, 2,065 participants aged 80–99 years, and 789 participants aged ≥100 years. The overall education level of the participants was low, with only 9.9% of participants having received more than 7 years of education. A total of 2,767 (63.2%) participants were residing in rural areas and 4,094 (93.5%) had a household income of < 100,000 yuan. Of them, 18.8% smoked, 17.5% drank alcohol, and 22.0% did physical activities. Participants with possible sarcopenia had lower BMI, waist circumference, calf circumference, grip strength, vitamin D3, and albumin (all *P* < 0.001). The mean of DDS was 5.14 ± 1.76. DDS in the possible sarcopenia group was significantly lower than that in the non-sarcopenia group (*P* < 0.001; Table 1).

**Table 1:** Baseline characteristics of all participants by sarcopenia status.

**Variables**	**Total sample *n* = 4,378**	**Possible sarcopenia *n* = 2,931**	**Non-sarcopenic *n* = 1,477**	***P***
**Sex (%)**				< 0.001
Men	2,006 (45.8)	1,225 (41.8)	781 (54.0)	
Women	2,372 (54.2)	1,706 (58.2)	666 (46.0)	
**Age group (year) (%)**				< 0.001
65–79	1,524 (34.8)	508 (17.3)	1,016 (70.2)	
80–99	2,065 (47.2)	1,668 (56.9)	397 (27.4)	
≥100	789 (18.0)	755 (25.8)	34 (2.4)	
**Marital status (%)**				< 0.001
Married	1,835 (41.9)	858 (29.3)	977 (67.5)	
Unmarried, divorced, or widowed	2,543 (58.1)	2,073 (70.7)	470 (32.5)	
**Education level (year) (%)**				< 0.001
0	2,655 (60.6)	2,094 (71.4)	561 (38.8)	
1–6	1,291 (29.5)	669 (22.8)	622 (43.0)	
≥7	432(9.9)	168 (5.7)	264 (18.2)	
**Pre-retirement occupation (%)**				< 0.001
Peasant	2,767 (63.2)	1,924 (65.6)	843 (58.3)	
No-peasant	1,611 (36.8)	1,007 (34.4)	604 (41.7)	
**Household income (yuan)**				0.048
≥100,000	284 (6.5)	175 (6.0)	109 (7.5)	
< 100,000	4,094 (93.5)	2,756 (94.0)	1,338 (92.5)	
**Smoking status (%)**				< 0.001
Previous	461 (10.5)	315 (10.7)	146 (10.1)	
Current	824 (18.8)	446 (15.2)	378 (26.1)	
Never	3,093 (70.6)	2,170 (74.1)	923 (63.8)	
**Consuming alcohol (%)**				< 0.001
Previous	329 (7.5)	216 (7.4)	113 (7.8)	
Current	768 (17.5)	438 (14.9)	330 (22.8)	
Never	3,281 (74.9)	2,277 (77.7)	1,004 (69.4)	
**Physical activities (%)**				< 0.001
Previous	120 (2.7)	90 (3.1)	30 (1.4)	
Current	961 (22.0)	530 (18.1)	431 (29.8)	
Never	3,307 (75.5)	2,311 (78.8)	996 (68.8)	
BMI (kg/m^2^)	21.78 ± 3.88	21.15 ± 3.84	23.07 ± 3.64	< 0.001
Waist circumference (cm)	81.69 ± 11.13	80.40 ± 10.92	84.31 ± 11.10	< 0.001
Calf circumference (cm)	29.90 ± 5.17	28.73 ± 4.90	32.26 ± 4.92	< 0.001
Grip strength max (kg)	18.88 ± 10.71	13.66 ± 7.19	29.46 ± 8.66	< 0.001
Total DDS	5.14 ± 1.76	4.82 ± 1.59	5.30 ± 1.54	< 0.001
**Total DDS quartile**				< 0.001
Q1 ( ≤ 4)	1,602 (36.6)	1,175 (40.1)	427 (29.5)	
Q2 (>4– ≤ 5)	866 (19.8)	593 (20.2)	273 (18.9)	
Q3 (5– ≤ 7)	1,543 (35.2)	972 (33.2)	571 (39.5)	
Q4 (≥8)	367 (8.4)	191 (6.5)	176 (12.2)	
Vitamin D3 (ng/L)	36.21 ± 19.45	34.79 ± 19.45	39.02 ± 19.15	< 0.001
Albumin (g/L)	42.55 ± 42.20	41.50 ± 41.00	44.62 ± 57.55	< 0.001

DDS, dietary diversity score; BMI, body mass index.

### 3.2. DDS and possible sarcopenia

**Figure 2:**
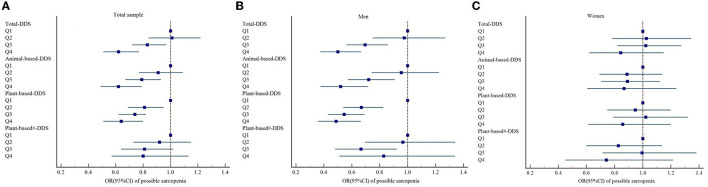
Forest plots of the association between DDS and possible sarcopenia based on Model 4 in **(A)** total sample, **(B)** men, and **(C)** women, respectively; Model 4: Adjusted for age, household income, education level, marital status, pre-retirement occupation, physical activities, smoking, drinking, BMI, waist circumference, vitamin D3, and albumin. DDS, dietary diversity score. ^#^A plant-based diet without legume products and nuts.

Based on the total DDS and classification of dietary sources of protein, GEE models were carried out to calculate the odds of possible sarcopenia. In the crude model, the participants who had the higher DDS (fourth quartile vs. the first quartile) showed lower odds of having possible sarcopenia, total diet (OR = 0.61; 95% CI: 0.52–0.72), animal-based diet (OR = 0.76; 95%CI: 0.67–0.87), plant-based diet (OR = 0.59; 95%CI: 0.49–0.71), and plant-based diet without legume products and nuts (OR = 0.49; 95% CI: 0.37–0.65) were significantly associated with decreased odds of possible sarcopenia. After adjustment for sex, age, household income, education level, marital status, and pre-retirement occupation in model 2, all associations were still significant. A further adjustment for physical activities, smoking, drinking, BMI, and waist circumference in model 3 revealed an inverse relationship between the odds of possible sarcopenia and total diet (OR = 0.66; 95% CI: 0.54–0.80), animal-based diet (OR = 0.63; 95% CI: 0.50–0.79), and plant-based diet (OR = 0.69; 95% CI: 0.55–0.86). No significant association was observed between a plant-based diet without legume products and nuts (OR = 0.81; 95% CI: 0.58–1.14) and possible sarcopenia. In the final model, which controlled all the confounding risk variables, total diet (OR = 0.62; 95%CI: 0.51–0.77), animal-based diet (OR = 0.62; 95% CI: 0.49–0.79), and plant-based diet (OR = 0.64; 95% CI: 0.51–0.80) were still associated with decreased odds of possible sarcopenia. A plant-based diet without legume products and nuts was not seemingly associated with possible sarcopenia (OR = 0.80; 95% CI: 0.57–1.13; Table 2 and Figure 2).

**Table 2:** Odds ratios of possible sarcopenia by quartiles of DDS among whole samples.

**Variables**	**Quartiles of DDS**				***P* trend**
	**Q1**	**Q2**	**Q3**	**Q4**	
**Total DDS**	(0, 4]	(4, 5]	(5, 7]	(7, 10]	
Model 1	1.00	0.89 (0.77–1.03)	0.78 (0.69–0.88)^*^	0.61 (0.52–0.72)^*^	< 0.001
Model 2	1.00	0.96 (0.80–1.15)	0.79 (0.68–0.91)^*^	0.60 (0.49–0.73)^*^	< 0.001
Model 3	1.00	1.01 (0.84–1.21)	0.84 (0.73–0.98)^*^	0.66 (0.54–0.80)^*^	< 0.001
Model 4	1.00	1.01 (0.84–1.22)	0.83 (0.72–0.97)^*^	0.62 (0.51–0.77)^*^	< 0.001
**Animal-based DDS**	[0, 1]	(1, 2]	(2, 3]	(3, 4]	
Model 1	1.00	0.82 (0.72–0.95) ^*^	0.76(0.67–0.87)^*^	0.76 (0.67–0.87)^*^	< 0.001
Model 2	1.00	0.84 (0.71–1.00)	0.72(0.62–0.87)^*^	0.57 (0.46–0.71)^*^	< 0.001
Model 3	1.00	0.90 (0.76–1.07)	0.77(0.66–0.91)^*^	0.63 (0.50–0.79)^*^	< 0.001
Model 4	1.00	0.91 (0.77–1.09)	0.79(0.67–0.93)^*^	0.62 (0.49–0.79)^*^	< 0.001
**Plant-based DDS**	(0, 2]	(2, 3]	(3, 4]	(4, 6]	
Model 1	1.00	0.79 (0.70–0.90)^*^	0.72 (0.63–0.83)^*^	0.59 (0.49–0.71)^*^	< 0.001
Model 2	1.00	0.81 (0.70–0.94)^*^	0.73 (0.62–0.86)^*^	0.62 (0.50–0.76)^*^	< 0.001
Model 3	1.00	0.85 (0.73–1.00)	0.77 (0.65–0.92)^*^	0.69 (0.55–0.86)^*^	< 0.001
Model 4	1.00	0.81 (0.69–0.95)^*^	0.74 (0.62–0.82)^*^	0.64 (0.51–0.80)^*^	< 0.001
**Plant-based**^**#**^ **DDS**	(0, 1]	(1, 2]	(2, 3]	(3, 4]	
Model 1	1.00	0.76 (0.64–0.92)^*^	0.60 (0.50–0.73)^*^	0.49 (0.37–0.65)^*^	< 0.001
Model 2	1.00	0.90 (0.72–1.12)	0.75 (0.60–0.94)^*^	0.68 (0.49–0.94)^*^	< 0.001
Model 3	1.00	0.96 (0.77–1.20)	0.83 (0.66–1.04)	0.81 (0.58–1.14)	0.018
Model 4	1.00	0.92 (0.73–1.15)	0.81 (0.64–1.02)	0.80 (0.57–1.13)	0.029

Model 1 included DDS as the sole variable; Model 2 further controlled for gender, age, household income, education level, marital status, and pre-retirement occupation; Model 3 additionally controlled for physical activities, smoking, drinking, BMI, and waist circumference; Model 4 added difficulty in vitamin D3 and albumin; DDS, dietary diversity score.

^#^A plant-based diet without legume products and nuts.

^*^p < 0.05.

### 3.3. Subgroup analyses

According to the subgroup analyses using the full model (model 4) by sex, associations were statistically significant for men but not for women. As shown in Table 3, the subgroup analyses by sex revealed that higher DDS of the total diet (OR = 0.50; 95% CI: 0.38–0.67), animal-based diet (OR = 0.52; 95% CI: 0.38–0.72), and plant-based diet (OR = 0.49; 95% CI: 0.38–0.66) was still associated with a decreased risk of possible sarcopenia in male participants (Figure 2).

**Table 3:** Association between quartiles of DDS and possible sarcopenia.

**Variable**	**Quartiles of DDS**				***P* trend**
	**Q1**	**Q2**	**Q3**	**Q4**	
**Men**				
Total DDS	1.00	0.98 (0.75–1.27)	0.70 (0.56–0.86)^*^	0.50 (0.38–0.67)^*^	< 0.001
Animal-based DDS	1.00	0.95 (0.74–1.22)	0.72 (0.57–0.91)^*^	0.52 (0.38–0.72)^*^	< 0.001
Plant-based DDS	1.00	0.67 (0.54–0.83)^*^	0.55 (0.43–0.69)^*^	0.49 (0.36–0.66)^*^	< 0.001
Plant-based^#^ DDS	1.00	0.97 (0.70–1.34)	0.67 (0.48–0.92)^*^	0.83 (0.51–1.34)	0.001
**Women**				
Total DDS	1.00	1.03 (0.78–1.34)	1.02 (0.82–1.27)	0.84 (0.62–1.15)	0.579
Animal-based DDS	1.00	0.89 (0.69–1.14)	0.89 (0.71–1.12)	0.87 (0.61–1.24)	0.317
Plant-based DDS	1.00	0.95 (0.75–1.20)	1.02 (0.79–1.32)	0.86 (0.61–1.20)	0.667
Plant-based^#^ DDS	1.00	0.83 (0.60–1.14)	0.99 (0.72–1.38)	0.74 (0.45–1.22)	0.813

The results were based on Model 4 controlling for age, household income, education level, marital status, pre-retirement occupation, physical activities, smoking, drinking, BMI, waist circumference, vitamin D3, and albumin; DDS, dietary diversity score.

^#^A plant-based diet without legume products and nuts.

^*^p < 0.05.

### 3.4. Sensitivity analyses

In a sensitivity analysis of the different DDSs, we obtained similar results using model 4. All the relationships between DDS (total diet, animal-based diet, plant-based diet, and plant-based diet without legumes products and nuts) and possible sarcopenia were consistent with model 4 in Table 2, after excluding participants who were long-term bedridden, had Alzheimer's disease, or were terminally ill (*n* = 118; Table 4).

**Table 4:** Sensitivity analysis of DDS with possible sarcopenia.

**Variables**	**Quartiles of DDS**				***P* trend**
	**Q1**	**Q2**	**Q3**	**Q4**	
Total DDS	1.00	1.01 (0.84–1.23)	0.84 (0.72–0.98)^*^	0.63 (0.51–0.77)^*^	< 0.001
Animal-based DDS	1.00	0.91 (0.76–1.08)	0.80 (0.68–0.94)^*^	0.62 (0.49–0.79)^*^	< 0.001
Plant-based DDS	1.00	0.81 (0.69–0.96)^*^	0.75 (0.63–0.89)^*^	0.65 (0.52–0.82)^*^	< 0.001
Plant-based^#^ DDS	1.00	0.91 (0.72–1.14)	0.81 (0.64–1.02)	0.81 (0.57–1.14)	0.043

The results were based on Model 4 by removing participants who were long-term bedridden, had Alzheimer's disease, or were terminally ill; Model 4: Adjusted for age, household income, education level, marital status, pre-retirement occupation, physical activities, smoking, drinking, BMI, waist circumference, vitamin D3, and albumin; DDS, dietary diversity score.

^#^A plant-based diet without legume products and nuts.

^*^p < 0.05.

## 4. Discussion

Based on the findings of this study, it was found that the total DDS, the animal-based DDS, and the plant-based DDS were significantly lower in Chinese older people with possible sarcopenia than in those people without sarcopenia. In sensitivity analyses, these associations between DDS and possible sarcopenia remained unchanged among older adults. According to subgroup analyses, the association was only found in men but not in women with significance. However, the association between a plant-based diet without legume products and nuts and possible sarcopenia was not marked.

Our findings support evidence from several previous studies among older adults that diet has a great influence on sarcopenia, including dietary variety, dietary quality, and dietary patterns. These different approaches for the study of the overall diet are complementary; thus, it is difficult to determine the best approach. Dietary patterns commonly strike a balance between the health benefits of food and individuals' dietary habits. The evidence from nutritional epidemiology suggests that the Mediterranean diet was positively associated with muscle function, such as slowing the decrease in walking speed and the risk of developing a new mobility disability (28). People with a high diet quality usually have more diversified eating habits. In a systematic review of diet quality and sarcopenia in developing economies, diet quality defined by diversity and nutrient adequacy was found to be positively associated with the components of sarcopenia (muscle mass, muscle strength, and physical performance) (27). Although dietary variety analyses do not necessarily take into account the “healthfulness” of foods within food groups as diet quality or patterns (such as Mediterranean diet), most dietary guidelines recommend adding a variety of foods. Several prospective studies provided strong support that a higher DDS leads to an increase in handgrip strength and general gait speed and that adherence to a diverse diet is associated with a healthier aging process in elderly people (23, 36).

We also observed some variations in the association between different DDSs and possible sarcopenia, while the associations between DDS (total diet, animal-based diet, and plant-based diet) and possible sarcopenia were more pronounced. When constructing a DDS, the food groups included were different across studies and may contribute to the inconsistent findings, partly due to the study population and purpose (21, 32, 33, 37). In the literature, the classification of DDS was based on the US Department of Agriculture (USDA) food guide pyramid (five food groups), the Chinese Dietary Guideline (10 food groups), and the Food and Agriculture Organization (FAO) guidelines (9 food groups) (32, 34, 38). In our study, we calculated the DDS based on the dietary guidelines for Chinese populations, which additionally includes some food groups which play important roles in the Chinese diet. We also referred to previous research on the food classification of an animal/plant-based diet. Research on dietary diversity can consider the intake of a variety of foods and nutrients at the same time, effectively avoided the limitations imposed by specific nutrients and food (39), and has more practical significance and popularization value.

In sarcopenia, several areas were identified as essential in terms of diet as follows: protein, vitamin D, calcium, folate, and antioxidants (4, 40). Dietary protein intake has been consistently reported to be positively associated with muscle mass, independent of study designs and populations (41, 42). Our results of the protein-based diet were consistent with previous studies that observed a significant association between high protein intake and a reduced prevalence of possible sarcopenia after classifying participants by protein dietary diversity. Even though animal protein was generally considered to be more effective at stimulating muscle protein synthesis than plant protein, meta-analyses demonstrated that changes in absolute lean mass and muscle strength were not affected by the protein source (16). Low intake of dietary protein including plant-based protein was associated with a higher risk of low muscle mass in older adults (43).

Plant protein quality is dependent on the food source and can have an equivalent nutritional value to that of animal protein. A recent meta-analysis concluded that soybean protein resulted in similar muscle mass and strength gains, similar to those of animal protein, on the basis of moderation and dietary variety (44). Protein quality depends on the amino acid composition and its capacity to be digested, absorbed, and utilized to meet the body's needs (16). Animal protein was considered to be of “high quality” as it provides adequate amounts of all the essential amino acids and tends to be well-digested (16). There was evidence to suggest that leucine may activate the signaling pathways leading to protein synthesis (4). Leucine and its metabolites, such as β-methyl butyrate and β-hydroxy, have been demonstrated as important constituents in the fight against sarcopenia (43). A high proportion of leucine was considered to be necessary to reverse suboptimal muscle protein synthesis and increase lean body mass in the elderly (4). Therefore, it is possible that the intentional consumption of proteinaceous food may mitigate the risk of factors associated with sarcopenia.

We also observed some variations in the associations by sex. The association between DDS (total diet, animal-based diet, and plant-based diet) and possible sarcopenia was only found to be statistically significant in older men. Hormonal decline associated with aging was also probable to impact the loss of muscle mass, with a reduction in testosterone and estrogen levels in women (4). Testosterone has been demonstrated to function in the development, maintenance, or rejuvenation of muscle tissues (45). Age-related declines of such hormones were observed in sarcopenia patients (46). Since the women in our research were older, it is likely that they may have lost more muscle because they had been postmenopausal for years and had lower sex hormones. These results reinforced the preventive role of diet intervention in the premenopausal period (47). Other studies also showed that the proper intake of meat in men and the sufficient intake of milk in women significantly reduced the risk ratio of sarcopenia (7).

In our study, a plant-based diet consisting only of grain-combined fruits, vegetables, and funguses was not significantly associated with possible sarcopenia. Previous research showed that fruits and vegetables also play a role in preventing sarcopenia, but no such associations were observed in women (29, 48). Fruits and vegetables, the primary source of antioxidants, may reduce the risk of sarcopenia due to the catabolic effects of oxidative stress on the skeletal muscle (48). It is uncertain whether the antioxidant effects of fruits and vegetables are limited. In addition, longitudinal analyses from different regions or countries did not always yield similar results due to differences in food availability and food preparation methods between regions (18). Previous studies have shown that diet and its diverse components may play an important role in regulating inflammation. The inflammatory potential of a diet have direct and indirect effects on low muscle mass, grip strength, and muscle mass, through one and the other, in aging (49). The consumption of ultra-processed foods is strongly associated with frailty risk in older adults (50). An anti-inflammatory diet may indirectly reduce the risk of physical disabilities or frailty, by slowing age-related sarcopenia and muscle wasting (50). Promoting an anti-inflammatory diet has the potential to prevent sarcopenia early in life. Further studies are required to clarify the relationship between different dietary diversity and sarcopenia.

Therefore, improvement of diet quality through a varied and adequate intake of protein may be essential for the prevention of sarcopenia. The interaction and mutual supplement of nutrients in a balanced diet play an important role in protecting body functions. For older adults, it is necessary to distinguish between men's and women's food. While some evidence suggested the benefits of healthier dietary patterns, the role of a non-exercise nutritional intervention in treating sarcopenia was much less clear. Questions about the appropriate intake of key nutrients (such as protein) and how these nutrients should be taken in terms of timing and distribution throughout the day remain controversial (1). The nutritional guidelines for treating and/or preventing sarcopenia, formulated by the Society for Sarcopenia, Cachexia, and Wasting Disease (SCWD), recommend a protein consumption of at least 1.0–1.5 g/kg BW/day in combination with appropriate exercise to prevent the loss of muscle mass and function with age (51). They point to the potential for sex effects that require further exploration.

Our study has some strengths. First, we used a large nationally representative sample of older adults in China for our study. We included a variety of covariates which allowed us to adjust for major potential confounders that were measured in the study population. Second, we utilized DDS which provides an overview of the diet and does not impose any restrictions based on specific nutrients or foods. Moreover, we performed a sensitivity analysis by excluding participants who were long-term bedridden, had Alzheimer's disease, or were terminally ill, and the results remained stable. Regarding the study limitations, we could not completely exclude memory bias since dietary data were self-reported and might not accurately reflect actual food intake. Furthermore, the dietary diversity in this study was based on frequency, which is a semi-quantitative measure, and it did not demonstrate a relationship between quantitative dietary diversity and outcomes. Additionally, the outcome indicators were part of epidemiological investigations, which may be subjected to a measurement bias. Due to the limitations of measurement conditions, equipment, and time, the measurement of calf circumference and grip strength (physical function) was completed in only a few eligible areas. Moreover, we excluded older adults with poorer health status and cognitive function since they may refuse or be unable to complete the survey and physical examination. Finally, only 6,624 cases were included. Although the fact that our study adjusted for a wide set of confounding factors, it was not possible to completely exclude residual and unmeasured confounding. Because of our cross-sectional study design, the study cannot conclude causality.

## 5. Conclusion

In summary, higher DDS was associated with a reduced risk of sarcopenia in the elderly. Adoption of a diverse diet may provide a cost-effective intervention for the prevention of sarcopenia. Prospective and/or intervention studies are necessary to analyze the causal relationship between dietary variety and the risk of sarcopenia.

## Data availability statement

Publicly available datasets were analyzed in this study. This data can be found here: https://doi.org/10.18170/DVN/WBO7LK.

## Ethics statement

The studies involving human participants were reviewed and approved by the Ethics Committee of Peking University, Peking University, Beijing, China. The patients/participants provided their written informed consent to participate in this study.

## Author contributions

YY, WZ, and XG contributed to the conception and design of the research. YL, FH, YZh, SL, CZ, and YZe contributed to data collection and acquisition. QD, XF, and HZ contributed to statistical analyses and interpretation. QD drafted the manuscript. YY, ZL, and XG revised the submitted version. All authors have read and agreed to approve the final manuscript.
